# Examining APOE4 overexpression effects in iAstrocytes across European and African ancestries

**DOI:** 10.1002/alz.092321

**Published:** 2025-01-03

**Authors:** Derek M. Dykxhoorn, Oded Oron, Jack Trittipo, Larry D. Adams, Takiyah D. Starks, Heriberto Acosta, Briseida E. Feliciano‐Astacio, Goldie S. Byrd, Margaret A Pericak‐Vance, Anthony J. Griswold, Jeffery M. Vance, Juan I Young

**Affiliations:** ^1^ Dr. John T. Macdonald Foundation Department of Human Genetics, University of Miami Miller School of Medicine, Miami, FL USA; ^2^ John P. Hussman Institute for Human Genomics, University of Miami Miller School of Medicine, Miami, FL USA; ^3^ Maya Angelou Center for Health Equity, Wake Forest University School of Medicine, Winston‐Salem, NC USA; ^4^ Carribean Center for the study of memory and cognition, San Juan, PR USA; ^5^ Universidad Central del Caribe, Bayamón, PR USA; ^6^ 1501 NW 10th Avenue, Miami, FL USA; ^7^ John P. Hussman Institute for Human Genomics, University of Miami Miller School of Medicine, Miami, FL, USA, Miami, FL USA; ^8^ John P. Hussman Institute for Human Genomics, Miami, FL USA; ^9^ John P. Hussman Institute for Human Genomics, Miller School of Medicine, Miami, FL USA

## Abstract

**Background:**

The *Apoliproprotein E (APOE) e4* allele is the most significant genetic risk factor for late‐onset Alzheimer disease (AD). However, the risk associated with the *APOE e4* allele differs across populations with individuals of African ancestry having a reduced risk than individuals of European (EU) ancestry. Further, single‐nuclei RNAseq analysis in autopsy samples from AD *APOEε4* homozygotes with EU Local Ancestry (LA) had a significantly increased *APOEε4* expression compared to those with African LA, particularly in astrocytes. To test the functional consequences of elevated APOE4 expression on astrocytes, we examined the effect of APOE4 overexpression in inducible pluripotent stem cell (iPSC) derived astrocytes (iAstrocytes).

**Method:**

iPSC were derived from European and African LA AD individuals homozygous for *APOEε4/ε4*. These *APOEε4/ε4* iPSC were differentiated into astrocytes (iAstrocytes) which were transduced with a lentiviral construct overexpressing *APOEε4* or a control vector. RNAseq analysis was performed to identify differentially expressed genes followed by pathway analyses.

**Result:**

iPSC lines were derived, validated for pluripotency, and differentiated into iAstrocytes. Preliminary analysis of transcriptional effects of*ApoEe4* overexpression in iAstrocytes showed alterations in gene expression patterns, including the down regulation of the inflammatory markers THBS1 and CLDN11 which had both been shown to be downregulated in AD, the mitochondrial‐related gene NDUFA4L2 shown to be dysregulated in AD, as well as SFRP2 (Wnt modulator involved in TREM2 processing). Upregulated genes included RGMA (accumulates in amyloid plaques), as well as TNC and CECR2. Pathway analysis showed an enrichment for extracellular matrix (ECM)‐receptor interaction, PI3K‐Akt signaling, and TGF‐beta signaling pathways. Interestingly, the transcriptional response to APOEε*4* overexpression has an ancestry‐specific component in these iAstrocytes; with some genes dysregulated exclusively in African ancestry iAstrocytes such as ZBTB4 and PRND, and others only in European iAstrocytes, including MYBPHL and VWF.

**Conclusion:**

The overexpression of *APOEε4* in iAstrocytes resulted in alteration in gene regulatory patterns, including the ECM and key signaling pathways across ancestries (EU and AF). In addition, gene expression differences were identified between ancestries. Further analyses of these ancestry‐specific transcriptional differences may help to elucidate the mechanisms that drive the differential AD risk seen in *APOEε4* individuals across ancestries.

